# Cutaneous Adverse Events Associated with Immune Checkpoint Inhibitors: A Review Article

**DOI:** 10.3390/curroncol29040234

**Published:** 2022-04-18

**Authors:** Chieh-Hsun Chen, Hsin-Su Yu, Sebastian Yu

**Affiliations:** 1 Department of Dermatology, Kaohsiung Medical University Hospital, Kaohsiung Medical University, Kaohsiung 807378, Taiwan; kitty84012918@gmail.com; 2 Graduate Institute of Clinical Medicine, College of Medicine, Kaohsiung Medical University, Kaohsiung 807378, Taiwan; yup.kmu@gmail.com; 3 Department of Dermatology, School of Medicine, College of Medicine, Kaohsiung Medical University, Kaohsiung 807378, Taiwan; 4 Neuroscience Research Center, Kaohsiung Medical University, Kaohsiung 807378, Taiwan

**Keywords:** immune checkpoint inhibitor, immune-related adverse event, cutaneous immune-related adverse event, anti-CTLA-4 inhibitor, anti-PD-1 inhibitor, anti-PD-L1 inhibitor

## Abstract

Immune checkpoint inhibitors (ICIs) have emerged as novel options that are effective in treating various cancers. They are monoclonal antibodies that target cytotoxic T-lymphocyte antigen 4 (CTLA-4), programmed cell death 1 (PD-1), and programmed cell death-ligand 1 (PD-L1). However, activation of the immune systems through ICIs may concomitantly trigger a constellation of immunologic symptoms and signs, termed immune-related adverse events (irAEs), with the skin being the most commonly involved organ. The dermatologic toxicities are observed in nearly half of the patients treated with ICIs, mainly in the form of maculopapular rash and pruritus. In the majority of cases, these cutaneous irAEs are self-limiting and manageable, and continuation of the ICIs is possible. This review provides an overview of variable ICI-mediated dermatologic reactions and describes the clinical and histopathologic presentation. Early and accurate diagnosis, recognition of severe toxicities, and appropriate management are key goals to achieve the most favorable outcomes and quality of life in cancer patients.

## 1. Introduction

In recent decades, immune checkpoints inhibitors (ICIs) have been demonstrated to dramatically improve the overall survival for a broad spectrum of advanced malignancies [1,2,3]. These agents are monoclonal antibodies that target the immune checkpoint molecules, including cytotoxic T-lymphocyte antigen 4 (CTLA-4), programmed cell death 1 (PD-1), and programmed cell death-ligand 1 (PD-L1). To date, seven ICIs have been approved by the U.S. Food and Drug Administration (FDA). Since March 2011, when the first immune checkpoint inhibitor, ipilimumab (an anti-CTLA-4 agent), was approved for the treatment of advanced (either metastatic or unresectable) melanoma [4], additional therapies that target the PD-1/PD-L1 axis have been subsequently approved, showing promising therapeutic outcomes for various solid tumors and hematologic malignancies [5]. Nivolumab, pembrolizumab, and cemiplimab are anti-PD-1 agents, whereas atezolizumab, durvalumab, and avelumab are anti-PD-L1 agents. The indications of these FDA-approved ICIs are summarized in Table 1. Other novel therapies targeting the alternative inhibitory pathways are currently under investigation, including lymphocyte-activation gene 3 (LAG-3), T-cell immunoglobulin and ITIM domain (TIGIT), T-cell immunoglobulin and mucin-domain containing-3 (TIM-3), V-domain immunoglobulin suppressor of T-cell activation (VISTA), B7 homolog 3 protein (B7-H3), inducible T cell costimulatory (ICOS), and B and T lymphocyte attenuator (BTLA) [6]. While ICIs promote the reinvigoration of the anti-tumor T-cell response, the enhanced immunologic activation may result in a variety of autoimmune-like or inflammatory side effects, termed immune-related adverse events (irAEs), which can involve almost any organ system.

## 2. Biologic Mechanism of Immune Checkpoint Inhibition

Immune checkpoint molecules, primarily of CTLA-4 and PD-1, are negative regulators adopted by cancer cells to disguise themselves as regular components of the human body, dampen the immune responses, and escape from the assault of human immunity [7,8]. An overview of the action of immune checkpoint inhibition gives insight into the anti-tumor function of the immune checkpoint blockades and the pathogenesis of irAEs.

In the physiologic state, immunologic inhibitory pathways are achieved through a complex network of costimulatory and inhibitory signals in order to maintain the immune response within a desired physiological range [8]. Both CTLA-4 and PD-1 are predominantly expressed on the T-lymphocytes. At the priming phase of T-cell activation, CTLA-4 primarily attenuates T-cell activity through competition with the costimulatory molecule CD28 for binding to the stimulatory receptors CD80 and CD86 expressed on the antigen-presenting cells (APCs) [8]. Moreover, the CTLA-4 expression on the regulatory T-cells (Tregs) mediates an inhibitory immune effect [8]. The main function of PD-1 is to attenuate local T-cell responses through interaction with PD-L1 and PD-L2 on APCs, ultimately inhibiting T-cell-receptor signaling in the periphery [8]. Tumors themselves, as well as the tumor microenvironment (TME), can express multiple inhibitory pathways and associated molecules, leading to T-cell impairment and immune escape. When these pathways are blocked by the immune checkpoint therapies, T-cell responses are promoted, facilitating an effective anti-tumor function and the exuberant activation of self-reactive T-cells with the resultant autoimmunity, mainly considered as irAEs [9].

## 3. Immune-Related Adverse Events (irAEs)

The spectrum of organ systems affected by irAEs is very broad, with varying frequencies and severities being reported [10,11,12]. The degree of severity and the associated morbidity of irAEs are graded according to the Common Terminology Criteria for Adverse Events (CTCAE), and the frequency of irAEs usually depends on the agents administered, the exposure time, the dosage, as well as the patient’s medical condition [12,13].

Among the irAEs encountered in ICI-treated patients, a systematic review has documented that all-grades colitis, hypophysitis, and rash were more frequent with anti-CTLA-4 agents, whereas pneumonitis, hypothyroidism, arthralgia, and vitiligo were more common with anti-PD-1 blockades [11]. De Velasco G. et al. [10] reported that the incidence of any-grade irAEs was higher in patients receiving an anti-CTLA-4 agent (53.8%) compared with those receiving anti-PD-1 (26.5%) or anti-PD-L1 (17.1%), whereas Martins F. et al. [12] demonstrated that the severe irAEs of grade III or IV were more frequently seen in ICIs targeting CTLA-4 (10 to 30%) than those targeting PD-1 (~10%). In theory, anti-CTLA-4 blockades act on the early stage of T-cell priming mainly in lymphatic organs, enhancing the magnitude of T-cell proliferation or reducing Treg-mediated immunosuppression, while anti-PD-1/PD-L1 blockades act on the later phase of the immune response mainly in the periphery, reinvigorating the pre-existing T-cell activity [14,15,16]. The dissimilar mechanisms of different target molecules may explain why anti-CTLA-4 inhibitors are associated with more prominent irAEs than anti-PD-1/PD-L1 inhibitors. In addition to single therapy, dual therapies with anti-CTLA-4 and anti-PD-1/PD-L1 blockades aim to further augment the therapeutic response but also increase the risk of irAEs [15,17,18,19]. In a phase III trial, combination therapy with ipilimumab and nivolumab demonstrated higher efficacy in treating metastatic melanoma compared with either monotherapy; however, treatment-related complications of grade III or IV were found in 55.0% of those in the nivolumab-plus-ipilimumab group, which is higher than that in the ipilimumab group (27.3%) and the nivolumab group (16.3%) [19]. Another study reported that combination therapy was associated with the highest risk of severe irAEs (55%), followed by patients receiving anti-CTLA-4 (10 to 41.6%) and anti-PD-1 (12 to 20%) as monotherapy [18]. Martins F. et al. [12] reported that the incidence of fatal toxicities is estimated to be approximately 0.3% to 1.3%, with a tendency to occur earlier (median onset time ~14.5 days) in the course of treatment and evolve rapidly, especially in patients with combination therapy.

## 4. Cutaneous Immune-Related Adverse Events (cirAEs)

Dermatologic complications arise as the earliest and most frequently observed adverse events among all irAEs, affecting between 30 and 50% of patients on ICIs [2,12]. The symptoms may significantly impair patients’ quality of life, and even lead to a pause of immunotherapy treatment. Fortunately, the majority of cirAEs seem to be mild and manageable, but there are still a few serious events (grade III or IV) being observed [17]. Maculopapular rash, pruritus, lichenoid eruptions, and vitiligo are the most widely reported cutaneous adverse events, which are summarized in Table 2 [17,20,21]. Severe cutaneous adverse reactions (SCARs), consisting of Stevens–Johnson syndrome/toxic epidermal necrolysis (SJS/TEN), drug rash with eosinophilia and systemic symptoms (DRESS), and acute generalized exanthematous pustulosis (AGEP), are rare but potentially life-threatening [17]. Other less-frequent manifestations include ICI-induced dermatomyositis, Sweet syndrome, interstitial granulomatous dermatitis, pityriasis rubra pilaris-like erythroderma, and lupus-like cutaneous reaction [22,23,24,25,26].

Previous studies have documented that the occurrence of certain cirAEs may indicate a more favorable clinical outcome for the treatment of underlying malignancies [20,27,28]. Teulings HE. et al. [27] reported that the development of vitiligo in patients with melanoma treated with immunotherapy implied improved progression-free and overall survival. In a retrospective study, the presence of lichenoid and spongiotic dermatitis could be an indicator of favorable oncologic outcomes in a small cohort of patients receiving anti-PD-1/PD-L1 [28]. In contrast, Han Y. et. al. [15] stated that cirAEs are dose-independent and agent-specific immune reactions, which might not be a prognostic indicator for improved outcomes. Moreover, there are several currently known biomarkers for predicting cirAEs. Jia XH et al. [29] stated that patients with a positive rheumatoid factor (RF) greater than 15 IU/mL prior to ICI treatment were more likely to develop immune-related dermatologic toxicities. Another prospective observational study found that HLA-DRB1*11:01 was significantly associated with pruritus, suggestive of a genetic etiology in cirAEs [30].

Given that numerous cirAEs share similar clinical manifestations to spontaneous autoimmune or inflammatory dermatoses, it is difficult to distinguish the concurrent dermatological disease from the cirAEs, particularly when there is a long period of latency between the initiation of ICI treatment and the onset of cirAEs [31]. Moreover, other concomitant medications can also contribute to the cutaneous adverse effects via allergic mechanisms, which may be similar to the presentation of cirAEs [32]. In a retrospective study, 80% of the patients who developed lichenoid eruptions after anti-PD-1/PD-L1 therapy were concurrently taking medications that have been previously reported to cause a lichenoid reaction [21].

**Table 2:** Summary of relatively common cutaneous adverse events associated with immune checkpoint inhibitors.

Cutaneous irAEs	Clinical Features	Histopathological Findings	Mainly Associated ICIs	Suggested Managements
Maculopapular eruption	Pruritic erythematous macules and papules coalescing into thin plaques, mostly on the trunk and extremities	Superficial, perivascular lymphocytes and eosinophils infiltrate into the upper dermis, mild epidermal spongiosis [33]	Anti-CTLA-4 > anti-PD-1/PD-L1	Symptomatic management with emollients, topical steroids, and oral antihistamines; consider systemic corticosteroids and withholding ICIs in severe cases [33]
Pruritus	May be concomitant with maculopapular rash or develop on normal- appearing skin	-	Anti-CTLA-4 > anti-PD-1/PD-L1	Topical emollients or oral antihistamines; consider topical/systemic corticosteroids or topical calcineurin inhibitors in severe cases; other therapies include aprepitant, doxepin, gabapentin, pregabalin, and naloxone [3,34,35]
Lichenoid dermatitis	Erythematous-to- violaceous scaly plaques with a localized or generalized distribution, mostly on the trunk and extremities; mucosal involvement is rarely reported	Hyperkeratosis, hypergranulosis, a sawtooth rete ridge pattern, lichenoid and interface lymphocytic infiltrates, basal vacuolar changes, parakeratosis, epidermal spongiosis and necrosis, and eosinophils may present [3,36,37,38]	Anti-PD-1/PD-L1	High-potency topical steroids; consider systemic corticosteroids and withholding ICIs in severe cases; other therapies include oral acitretin and phototherapy [39,40,41]
Psoriasiform dermatitis	Sharply bordered, scaly, and erythematous plaques, mostly at extensor sites	Hyperkeratosis, hypogranulosis, acanthosis with elongated rete ridges, perivascular lymphocytic infiltration [42,43]	Anti-PD-1/PD-L1	Topical corticosteroids, topical vitamin D analogs, or topical retinoids; phototherapy (NB-UVB) [44]; other therapies include acitretin, apremilast, and methotrexate [45]; biologic agents and systemic steroids should be carefully used (TNF-α inhibitors are contraindicated)
Vitiligo-like depigmentation (VLD)	Multiple depigmented flecked lesions coalescing into patches on photoexposed areas; the Koebner phenomenon (-)	Dermal lymphocytic infiltrates and a lack of melanocytes [46]	Anti-PD-1/ PD-L1 > anti-CTLA-4	No effective treatment
Bullous pemphigoid (BP)	Pruritic tense bullae overlying the urticarial plaques, mostly on the trunk and extremities	A subepidermal cleft with numerous eosinophils; DIF shows a linear deposition of C3 and IgG along the basement membrane zone	Anti-PD-1/PD-L1	High-potency topical steroids or systemic corticosteroids depending on the extent of disease; other therapies include methotrexate, doxycycline, omalizumab, and rituximab [47,48,49,50,51]
SJS/TEN	Flaccid blister formation (Nikolsky’s sign +) and rapidly progressive and extensive epidermal necrosis and desquamation; mucosal involvement is common	Full-thickness epidermal necrolysis with extensive keratinocyte necrosis, subepidermal bullae, and dermal infiltrates with lymphocytes, eosinophils, and neutrophils	Anti-CTLA-4 > anti-PD-1/PD-L1	Permanent cessation of ICIs, high-dose systemic corticosteroids and IVIG; intense supportive care (keeping a balance of electrolytes, fluid, and nutrition) and wound care; other therapies include TNF-α inhibitors, mycophenolate mofetil, cyclosporin, and plasmapheresis [52,53,54,55]

Abbreviations: irAEs, immune-related adverse events; SJS/TEN, Stevens–Johnson syndrome/toxic epidermal necrolysis; DIF, direct immunofluorescence; ICIs, immune checkpoint inhibitors; anti-CTLA-4, anti-cytotoxic T-lymphocyte antigen 4; anti-PD-1, anti-programmed cell death 1; anti-PD-L1, anti-programmed cell death-ligand 1; NB-UVB, narrowband ultraviolet B; TNF-α, tumor necrosis factor-alpha.

### 4.1. Maculopapular Eruption (Eczema-like Dermatitis)

**Figure 1:**
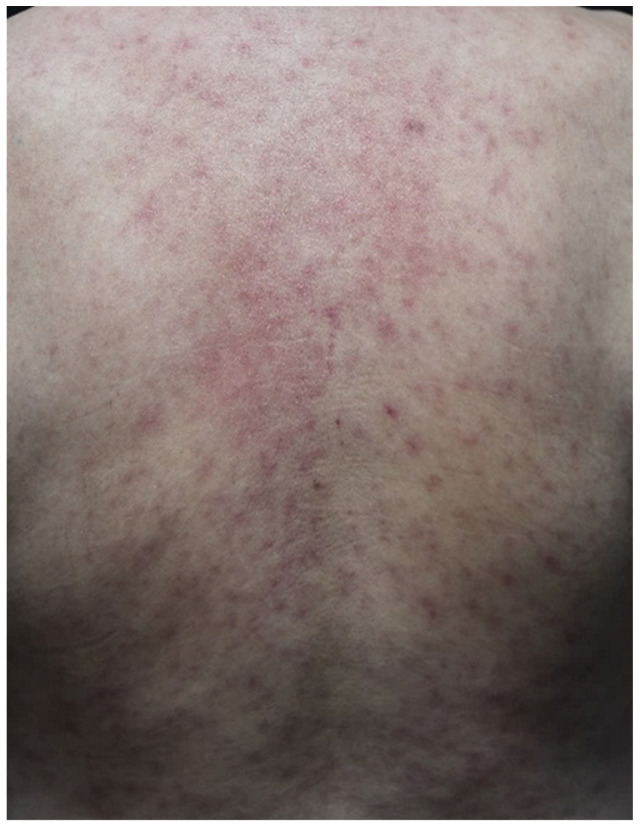
Maculopapular eruption. Diffuse, asymptomatic, erythematous maculopapular rash on the trunk and four extremities in a patient with hepatocellular carcinoma who started atezolizumab treatment 15 days prior.

The development of maculopapular rash is observed in approximately 49% to 68% of patients receiving anti-CTLA-4 agents, compared with 20% of patients receiving anti-PD1/PDL-1 therapy, and the eruption usually occurs within the first 3 to 4 weeks after the initiation of ICI therapy [33,56,57]. The clinical presentation is relatively nonspecific and characterized by pruritic erythematous macules and papules coalescing into thin plaques, with the trunk and extremities mainly affected (Figure 1). The lesions usually spare the face, palms, and soles. In some cases, it appears as an exacerbation of a pre-existing skin condition, such as eczema or rosacea [33]. Histologically, superficial, perivascular lymphocytes and eosinophils infiltrate into the upper dermis, and mild epidermal spongiosis can be present [33].

It is important to be aware that on rare occasions, a maculopapular eruption may be the initial presentation of bullous pemphigoid (BP), SJS/TEN, or DRESS, which require a close follow-up [39]. The patient should be carefully assessed for the appearance of blister formation, mucosal involvement, skin pain, fever, lymphadenopathy, or erythroderma [39]. Laboratory investigation and skin biopsy should be considered if there is an evolution of lesions or the development of any concerning symptom or sign.

Treatments mainly include symptomatic management, and pruritus can be managed with emollients, topical steroids, and oral antihistamines. Since the symptoms are usually mild and self-limiting with resolution within 2 to 3 months, interruption or discontinuation of ICIs is not always necessary [33]. However, severe cases (grade III or above) may require systemic steroids and withholding ICI therapy.

### 4.2. Pruritus

Pruritus is among the most prevalent cutaneous adverse reactions to ICI therapy, with its all-grade incidence ranging from 13 to 20% with nivolumab and pembrolizumab, respectively [28,58,59]. A higher incidence was reported in patients treated with anti-CTLA-4 agents (25–36%), and the highest incidence was reported in patients treated with combination therapy (33–47%) [58]. Although it is typically concomitant with maculopapular rash, it can precede it or develop independently on normal-appearing skin. Symptoms are usually mild-to-moderate in severity (grade I or II), but high-grade pruritus occurs in less than 1% of patients and can severely impair the quality of life [2,59,60].

The treatment depends on the severity of the pruritus. Mild cases may respond to topical emollients or first-generation antihistamines, while topical or systemic glucocorticoids or topical calcineurin inhibitors should be considered in more severe cases [3,34]. The efficacy of aprepitant has been described in a Japanese patient with severe refractory pruritus during nivolumab treatment [61]. Additional medications, including doxepin, gabapentin, pregabalin, and naloxone, have been documented [3,34,35].

### 4.3. Lichenoid Dermatitis

**Figure 2:**
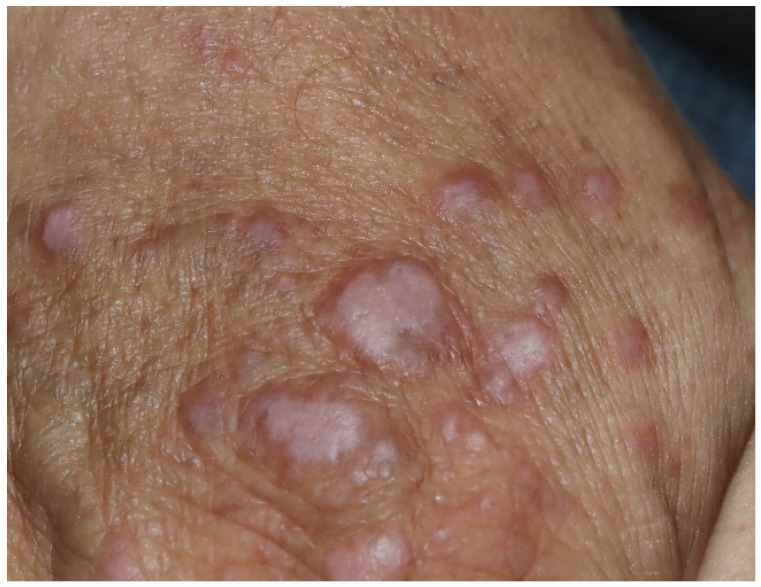
Lichenoid dermatitis. Scattered pruritic, violaceous-to-erythematous, flat-topped scaly papules and plaques on the scalp, face, bilateral dorsal hands, and anterior chest, with a predilection for the sun-exposed area, in a patient with lung cancer receiving atezolizumab.

Lichenoid drug eruptions are reported to be relatively common among cirAEs and occur more frequently in patients receiving anti-PD-1/PD-L1 blockades than in patients receiving anti-CTLA-4 blockades [36,37,39,60,62]. Lichenoid dermatitis affects nearly one-fifth of patients treated with anti-PD-1 agents, and the time to onset of lichenoid dermatologic toxicity ranges from 3 days to 13 months from the initiation of anti-PD-1 therapy [21,38]. The clinical presentation is characterized by erythematous-to-violaceous scaly plaques in a variety of distributions, with either discrete papules or plaques in a localized area or a more generalized distribution with a predilection for the trunk and extremities (Figure 2) [21,32]. While cutaneous lichenoid reactions have emerged as common side effects, involvement of the oral mucous membrane is rarely described [36,63,64,65]. In a case series, oral lichenoid eruptions were documented in 10 cases treated with pembrolizumab, nivolumab, or atezolizumab, whereas another report presented two cases developing ulcerative oral lichenoid reactions after nivolumab treatment [64,65]. Other clinical variants, including inverse presentation, bullous lichen planus pemphigoid, and erosive and hypertrophic variants, have been documented [38,66,67,68]. The pathologic features are similar to lichen planus, with the presence of hyperkeratosis, hypergranulosis, a saw-tooth rete ridge pattern, lichenoid and interface lymphocytic infiltrates, and basal vacuolar changes [36,37]. However, unlike typical lichen planus, parakeratosis, epidermal spongiosis and necrosis, and eosinophils may be present [3,36,37,38].

The treatment initially consists of high-potency topical steroids, and in most of the cases, interruption of ICI therapy is not necessary, while systemic corticosteroids and cessation of ICI therapy may be required in cases of high-grade toxicity. Alternative therapies for severe cases include oral acitretin and phototherapy, which were both reported to be effective [39,40,41]. It is also important to note that erosive oral or genital lichenoid reactions should be treated aggressively with systemic retinoids or oral prednisolone due to their scarring potential [39].

### 4.4. Psoriasiform Dermatitis

Psoriasiform dermatitis can be either de novo or a flare-up of pre-existing psoriasis in patients treated with anti-PD-1/PD-L1, and approximately 3% of patients in Japan treated with nivolumab developed psoriasis-like reactions [42,69,70,71,72]. In a study of 21 patients, the average duration between anti-PD1 initiation and psoriasis flare-up was about 50 days, which is a shorter duration than that of de novo psoriasiform eruptions (91 days) [69]. The typical presentation is plaque psoriasis, although guttate, pustular, inverse, and palmoplantar variants have been less frequently described [69,70,71]. The skin lesions are characterized by sharply bordered, erythematous scaly plaques, mostly at localized extensor sites. The histopathological features are similar to typical psoriasis vulgaris, with the presence of hyperkeratosis, hypogranulosis, acanthosis with elongated rete ridges, and a perivascular lymphocytic infiltration [42,43].

The immune mechanisms of ICI-mediated psoriasiform eruptions remain uncertain. In murine models, PD-1 blockade, either by a genetic deficiency or monoclonal antibody treatment, was found to enhance the production of interleukin (IL)-17A and IL-22 by activated γδ-low (GDL)-expressing T cells, promote neutrophil infiltration into the epidermis, and thereby induce psoriasiform skin inflammation [44,72,73,74].

Management should be carried out by applying a multidisciplinary approach. The initial treatment includes topical corticosteroids, topical vitamin D analogs, or topical retinoids. Phototherapy with narrowband ultraviolet B (NB-UVB) light may be helpful when used in conjunction [44]. Systemic options may be considered when topical treatment is ineffective. In a multicentric study of 115 European patients, acitretin, apremilast, and methotrexate were found to be efficacious and safe options for ICI-mediated psoriasis [45]. Biologic agents, particularly tumor necrosis factor (TNF)-α inhibitors, are contraindicated since they may promote the occurrence and progression of cancers [3,39,75,76]. IL-12/23 inhibitors, such as ustekinumab, act upstream of the immune signaling and may carry a higher risk of infection due to immunosuppression [39,77]. However, biologic agents targeting IL-23 or IL-17 may be considered in severe or recalcitrant cases given their selective inhibition of the T helper 17 (Th17) axis in psoriasis, minimal immunosuppressive effect, and rapid onset of action [39,78]. It is also important to note that, similar to spontaneous psoriasis, systemic steroids should be carefully prescribed, since they may carry the risk of a severe rebound of psoriasis upon steroid withdrawal [3,39,75].

### 4.5. Vitiligo-like Depigmentation (VLD)

Vitiligo-like depigmentation (VLD) appears most frequently in patients treated for melanoma, although other cancers have rarely been reported [46,79,80,81]. In a retrospective study in Italy, VLD was induced by anti-CTLA-4 inhibitors, anti-PD-1 inhibitors, and the combination therapy in 32%, 56%, and 12% of patients, respectively, with a median onset time of around 26 weeks [82]. Larsabal M. et al. [83] reported that ICI-induced vitiligo is distinct from idiopathic vitiligo in that it consists of multiple flecked lesions coalescing into patches on the photoexposed areas, and it is not associated with the Koebner phenomenon. The histologic features of VLD include an inflammatory infiltrate in the dermis with a predominance of T cells and a lack of melanocytes [3,46]. Immunotherapy-induced vitiligo potentially corresponds to a cross-reaction against melanocyte differentiation antigens (MART-1, gp100, and tyrosinase-related proteins 1 and 2) shared by healthy and malignant melanocytes, and cytotoxic T lymphocytes are thought to be the main effector cells that recognize these shared antigens, which were found to infiltrate both tumor and vitiligo tissues [46,84].

There is no definite treatment for ICI-induced vitiligo, and most of the cases with VLD do not resolve after discontinuation of ICIs [3]. Photoprotection with sunscreen and clothing should be encouraged to avoid sunburns, and camouflaging can be performed to limit the psychosocial impact [3,46]. Moreover, the occurrence of VLD in patients treated for melanoma may represent a positive prognostic factor, with a favorable response and prolonged overall survival [27,46,85].

### 4.6. Bullous Pemphigoid (BP)

**Figure 3:**
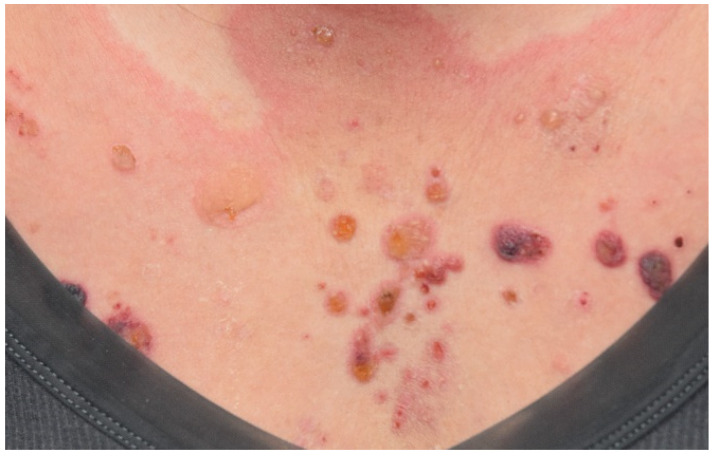
Bullous pemphigoid. Multiple pruritic tense bullae with erosions on the background of urticarial patches on the trunk and four extremities in a patient with metastatic lung cancer treated with nivolumab.

Compared with other dermatoses, immunobullous disorders are relatively rarely reported in the literature, with most associated with anti-PD-1/PD-L1 blockades [47,68,86,87,88]. In a retrospective analysis including 853 patients receiving anti-PD-1/PD-L1, the incidence of bullous skin toxicity was approximately 1%, with bullous pemphigoid (BP) appearing to be the most common presentation, followed by bullous lichenoid dermatitis and linear IgA bullous dermatosis [47]. The clinical manifestation of BP is usually characterized by pruritic, tense bullae overlying the urticarial plaques mainly on the trunk and extremities (Figure 3); however, urticarial-like or eczematous rash may be the prodromal presentation or the “non-bullous” variants [89,90]. Involvement of the mucosal membrane is less frequent [48,63].

In addition to serologic investigations, the standard diagnostic work-up for bullous diseases comprises a dermatologic referral and biopsy specimens for initially establishing whether the site of splitting is intraepidermal or subepidermal [87]. Further assessments including direct immunofluorescence (DIF) and indirect immunofluorescence (IIF) are also necessary [87]. The histopathologic features are similar to those of classic BP, which include a subepidermal cleft with numerous eosinophils and linear deposition of complement component 3 (C3) and immunoglobulin G (IgG) along the basement membrane zone on DIF [87].

A number of theories have been developed to explain the immunologic mechanism of ICI-related BP. It is evident that BP is mediated by autoantibodies against BP180, the hemidesmosomal proteins that are expressed both on certain tumor cells (such as melanoma and non-small cell lung carcinoma) and the basement membrane of the skin [87,91]. In anti-PD-1/PD-L1-induced BP, it is possible that the reinvigoration of the T-cell response targets BP180 on cancer cells, as well as the basement membrane of the skin, thereby inducing BP [87].

As for the treatment strategy for ICI-induced BP, a mild presentation (grade I, < 10% body surface area (BSA)) may respond to high-potency topical steroids, whereas patients with more extensive (grade II and above, >10% BSA) eruptions or with mucosal involvement may require systemic corticosteroids as well as the interruption of ICI therapy, either temporary or permanent [47]. Other steroid-sparing agents include methotrexate, doxycycline with or without nicotinamide, and omalizumab, which are reported to be effective therapies [47,48,49,50]. Interestingly, in contrast to classic BP, which typically resolves upon discontinuation of the offending agent, ICI-induced BP may persist for several months after the cessation of the causative agent owing to prolonged immune activation [32,59,87]. The administration of rituximab, an anti-CD20 monoclonal antibody, may be considered in severe or recalcitrant cases, and successful use has been demonstrated in the literature [51,92,93,94].

### 4.7. Stevens–Johnson Syndrome (SJS)/Toxic Epidermal Necrolysis (TEN)

Severe cutaneous adverse reactions (SCARs), consisting of Stevens–Johnson syndrome/toxic epidermal necrolysis (SJS/TEN) and drug reaction with eosinophilia and systemic symptoms (DRESS), are rare dermatologic toxicities that can be potentially life-threatening and, hence, should be managed aggressively [52,95,96,97]. The occurrence of SCARs is related to both anti-CTLA-4 and anti-PD1/PD-L1 blockades, with the latency periods varying from 5 to 91 days [97,98]. In SJS/TEN, the constitutive symptoms, including fever, anorexia, and malaise, are followed by skin eruptions of flaccid blister formation with a positive Nikolsky’s sign and rapidly progressive and extensive epidermal necrosis and desquamation. Mucosal involvement of the oral tract, gastrointestinal tract, respiratory tract, and genitalia may occur [99,100]. It is important to note that nonspecific morbilliform eruptions may precede the severe drug reactions; therefore, the careful monitoring of patients with morbilliform rash is necessary to assess a possible evolution [39,95]. Biopsy specimens typically reveal full-thickness epidermal necrolysis with extensive keratinocyte necrosis, subepidermal bullae, and varying degrees of inflammation containing lymphocytes, eosinophils, and neutrophils in the superficial dermis.

In these severe cases, permanent ICI cessation is necessary. The mainstay of management requires intense supportive care ensuring the homeostasis of fluid and electrolytes, as well as minimizing the infectious risks with wound care and topical or systemic antibiotics treatment. High-dose systemic corticosteroids (methylprednisolone at 1 to 2 mg/kg/day) and intravenous immunoglobulin (IVIG) should be administered [52,53]. Additional medications, such as TNF-α inhibitors (infliximab or etanercept), mycophenolate mofetil, or cyclosporin, may be considered [52,53,54]. Plasmapheresis can be used in some cases [39,55].

### 4.8. Other Less-Common cirAEs

Other less-common cutaneous immune-related adverse events reported as case reports or case series are summarized in Table 3.

## 5. Conclusions

Owing to the increasingly widespread use of ICI therapy in cancer treatment, a better understanding of irAEs is warranted. Both physicians and patients should be well-educated about these adverse events. Maculopapular eruption, pruritus, and SJS/TEN are more commonly seen with anti-CTLA-4 blockades, whereas lichenoid dermatitis, psoriasiform dermatitis, vitiligo-like depigmentation, and bullous pemphigoid more frequently occur in patients treated with anti-PD-1/PD-L1 blockades. While the majority of cutaneous adverse reactions are usually mild, severe cirAEs such as STS/TEN and DRESS are life-threating and require the cessation of ICIs. Early and accurate diagnosis, recognition of severe adverse effects, as well as appropriate management are key goals in treating patients receiving ICIs. It is important that a multidisciplinary team involving oncologists and dermatologists be engaged in the assessment of cirAEs, providing patients with better care and an important opportunity to continue to benefit from the anti-tumor treatment.

## Figures and Tables

**Table 1:** Summary of immune checkpoint inhibitors approved by the Food and Drug Administration.

ICIs	Target	Indications
Ipilimumab	CTLA-4	CRC, HCC, melanoma, mesothelioma, NSCLC, RCC
Nivolumab	PD-1	CRC, esophageal SCC, HCC, HL, HNSCC, melanoma, mesothelioma, NSCLC, RCC, urothelial carcinoma
Pembrolizumab	PD-1	breast cancer, cervical cancer, CRC, CSCC, endometrial carcinoma, esophageal carcinoma, gastric carcinoma, HCC, HL, HNSCC, melanoma, mesothelioma, MCC, NSCLC, large B-cell lymphoma, RCC, SCLC, urothelial carcinoma
Cemiplimab	PD-1	BCC, CSCC, NSCLC
Atezolizumab	PD-L1	breast cancer, HCC, melanoma, NSCLC, SCLC, urothelial carcinoma
Durvalumab	PD-L1	NSCLC, SCLC, urothelial carcinoma
Avelumab	PD-L1	MCC, RCC, urothelial carcinoma

Abbreviations: ICIs, immune checkpoints inhibitors; CTLA-4, cytotoxic T-lymphocyte antigen 4; CRC, colorectal cancer; PD-1, programmed cell death 1; HCC, hepatocellular carcinoma; NSCLC, non-small cell lung cancer; RCC, renal cell carcinoma; SCC, squamous cell carcinoma; HL, Hodgkin’s lymphoma; HNSCC, head and neck squamous cell carcinoma; CSCC, cutaneous squamous cell carcinoma; MCC, Merkel cell carcinoma; SCLC, small cell lung cancer; BCC, basal cell carcinoma; PD-L1, programmed cell death receptor-1 ligand.

**Table 3:** Other less-common dermatologic toxicities associated with immune checkpoint inhibitors.

Less-Common cirAEs	Description	Suggested Managements
Alopecia areata/ universalis [101,102,103]	The incidence of alopecia was 1.0 to 2.0% of patients treated with ICIs [103]. Regrowing hair may exhibit poliosis and the texture of hair may change [101,104].	Intralesional triamcinolone or topical corticosteroids Topical DPCP [105] or SADBE [106] Systemic corticosteroids may be considered in alopecia universalis
Sarcoidosis/ sarcoidosis-like reactions [107,108,109]	Sarcoidosis-like reactions are most related to ipilimumab, with the time to onset ranging from 3 weeks to 2 years [107]. A multisystem disease characterized by granulomas in various organs; involvement in the lungs, hilar and mediastinal lymph nodes, and skin is frequent.	Dependent on the extent of Involvement. Topical or intralesional corticosteroids for only cutaneous involvement Systemic corticosteroids for systemic involvement ICIs may be reintroduced after the resolution of sarcoidosis
Erythema nodosum (EN) [109,110,111,112]	The lesions present as painful erythematous nodules, most commonly on the anterior aspects of the lower extremities, that may be accompanied by fever and arthralgia.	Topical corticosteroids, NSAIDs, and continuation of ICIs for mild cases Systemic corticosteroids and cessation of ICIs for severe cases or with systemic symptoms
Sweet syndrome [26,113,114,115]	The latency period between Sweet syndrome onset and the first dose of ipilimumab was found to be 6 to 12 weeks [113]. Clinical manifestations include fever and an abrupt eruption of painful, erythematous papules, plaques, and nodules. Sweet syndrome responds rapidly to oral corticosteroids.	Systemic corticosteroids (prednisolone at 0.5 to 1 mg/kg/day) Dapsone or colchicine may be considered as steroid-sparing agents
Pyoderma gangrenosum (PG) [113,116,117,118]	Ipilimumab-related PG occurred 16 weeks after ICI initiation [113]. The lesion starts as a small pustule or red bump and then breaks down, resulting in a central ulcer with erythematous undermined borders.	Topical, oral, or intralesional corticosteroids Wound care, pain management, and topical antibiotics for preventing infection Dapsone, colchicine, and minocycline may be potential treatment options
Dermatomyositis (DM) [22,119,120,121]	DM may present as either a drug-induced reaction or a paraneoplastic phenomenon. The time course of disease development and an anti-TIF1-γ antibody titer can help to identify this disorder [120]. DM is characterized by proximal muscle weakness and typical skin lesions, including heliotrope rash, Gottron’s papules, and photodistributed erythema. The serologic testing may show elevated CK, CRP, and ESR, or findings can be normal. Anti-Jo-1 was usually negative in this setting [31,58].	Cessation of ICI therapy Systemic corticosteroids Topical corticosteroids for involved skin Additional treatments include azathioprine, methotrexate, tacrolimus, or IVIG (1 g/kg/day)
Grover’s disease (GD) [122,123]	Grover’s disease typically presents as an intensely pruritic, papulovesicular eruption, mostly on the central back, mid chest, and upper arms. The skin rash and pruritus may persist for months after ICI disruption [123].	The first-line therapy includes topical emollients, topical corticosteroids, or oral antihistamines for relieving pruritus In severe or persistent cases, systemic corticosteroids or topical/oral retinoids can be used Phototherapy
Drug reaction with eosinophilia and systemic symptoms (DRESS) [124,125]	DRESS is a phenotype of SCARs and can be potentially fatal with a mortality rate of up to 10% [98]. DRESS is a type IV hypersensitivity reaction. It is typically characterized by fever, skin involvement with generalized maculopapular exanthem, facial edema, lymphadenopathy, internal organ involvement, and hematologic abnormalities (atypical lymphocytosis and eosinophilia).	Cessation of ICI therapy A moderate-to-high dose of systemic corticosteroids (prednisolone at 0.5 to 1 mg/kg/day or equivalents) with a slow taper Supportive care and gentle skin care with emollients
Acute generalized exanthematous pustulosis (AGEP) [126,127,128,129]	AGEP is a phenotype of SCARs that is relatively benign, but ~4% of patients can develop a fatal situation [98]. The latency period between AGEP onset and ICI initiation was found to be 3 to 12 weeks [113]. The lesions present as an eruption of numerous nonfollicular sterile pustules overlying the edematous erythema with systemic involvement, such as fever and neutrophilia.	Cessation of ICI therapy Systemic corticosteroids Symptomatic management with moisturizers, topical corticosteroids, and oral antihistamines

Abbreviations: cirAEs, cutaneous immune-related adverse events; ICIs, immune checkpoint inhibitors; DPCP, diphenylcyclopropenone; SADBE, squaric acid dibutylester; NSAID, non-steroidal anti-inflammatory drug; anti-TIF1-γ antibody, anti-transcription intermediary factor 1-gamma antibody; CK, creatine kinase; CRP, c-reactive protein; ESR, erythrocyte sedimentation rate; IVIG, intravenous immunoglobulin; SCARs, severe cutaneous adverse reactions.

## Data Availability

Not applicable.
